# Inhibition of prefrontal protein synthesis following recall does not disrupt memory for trace fear conditioning

**DOI:** 10.1186/1471-2202-7-67

**Published:** 2006-10-06

**Authors:** Sonja Blum, Jason D Runyan, Pramod K Dash

**Affiliations:** 1 The Vivian L. Smith Center for Neurologic Research and Department of Neurobiology and Anatomy, The University of Texas, Houston, Texas 77225, USA

## Abstract

**Background:**

The extent of similarity between consolidation and reconsolidation is not yet fully understood. One of the differences noted is that not every brain region involved in consolidation exhibits reconsolidation. In trace fear conditioning, the hippocampus and the medial prefrontal cortex (mPFC) are required for consolidation of long-term memory. We have previously demonstrated that trace fear memory is susceptible to infusion of the protein synthesis inhibitor anisomycin into the hippocampus following recall. In the present study, we examine whether protein synthesis inhibition in the mPFC following recall similarly results in the observation of reconsolidation of trace fear memory.

**Results:**

Targeted intra-mPFC infusions of anisomycin or vehicle were performed immediately following recall of trace fear memory at 24 hours, or at 30 days, following training in a one-day or a two-day protocol. The present study demonstrates three key findings: 1) trace fear memory does not undergo protein synthesis dependent reconsolidation in the PFC, regardless of the intensity of the training, and 2) regardless of whether the memory is recent or remote, and 3) intra-mPFC inhibition of protein synthesis immediately following training impaired remote (30 days) memory.

**Conclusion:**

These results suggest that not all structures that participate in memory storage are involved in reconsolidation. Alternatively, certain types of memory-related information may reconsolidate, while other components of memory may not.

## Background

Protein synthesis dependency around the time of training is a hallmark of long-term memory consolidation [1]. This has been repeatedly demonstrated across species and types of new information learned [2]. Reactivation of information already committed to long-term memory may induce an additional protein synthesis dependent period, during which the original memory can be disturbed by administration of protein synthesis inhibitors, suggestive of a reconsolidation process [3], although alternative interpretations have been suggested [4]. While protein synthesis dependencies have been observed for both consolidation and reconsolidation, evidence suggests that consolidation and reconsolidation differ in several key aspects (for review see [5])[6]. For example, a protein synthesis-dependent period follows every training paradigm (consolidation), whereas *not *every reactivation of memory results in a re-dependency on protein synthesis (reconsolidation). Whether a reactivated memory undergoes reconsolidation appears to depend on several factors, including the age of the memory and training intensity [7,8] For example, a conditioned taste aversion (CTA) memory resulting from a more intense, two day, training protocol is susceptible to reconsolidation, whereas a one day training paradigm results in a memory susceptible to extinction [8]. An additional key difference is that not all brain regions involved in consolidation are involved in reconsolidation. For example, in inhibitory avoidance, protein synthesis in the dorsal hippocampus is required for consolidation but not reconsolidation of memory [9]. Similarly, in CTA, consolidation depends on protein synthesis in the central nucleus of the amygdala, whereas there is no renewed protein synthesis dependency in this region following memory reactivation [10]. In young chicks, following a reminder, expression of the transcription factor c-Fos is seen only in one of the two regions involved in consolidation [11], further suggesting that not all structures involved in consolidation participate in reconsolidation.

Trace fear conditioning is a form of learning that involves the association between a tone (conditioned stimulus, CS) and a footshock (unconditioned stimulus, US) that are separated by a time gap (trace period). The hippocampus and the medial prefrontal cortex (mPFC) are required for making the CS-US association, and both are required for consolidation of memory for trace fear conditioning [12-17] We have recently reported that, in addition to its role in consolidation of trace fear memory, the hippocampus is a site of renewed protein synthesis dependency following trace fear memory reactivation [18]. Consistent with previous reports, only a more intense (two day) training protocol resulted in a memory susceptible to reconsolidation, whereas a one day training resulted in a memory susceptible to extinction. In the present study, we examine whether the mPFC is also a site of protein synthesis dependent reconsolidation following reactivation of trace fear memory for one- or two-day training paradigms.

## Results

### Inhibition of prefrontal protein synthesis following recall of a one-day trace fear conditioning paradigm does not disrupt memory

**Figure 1:**
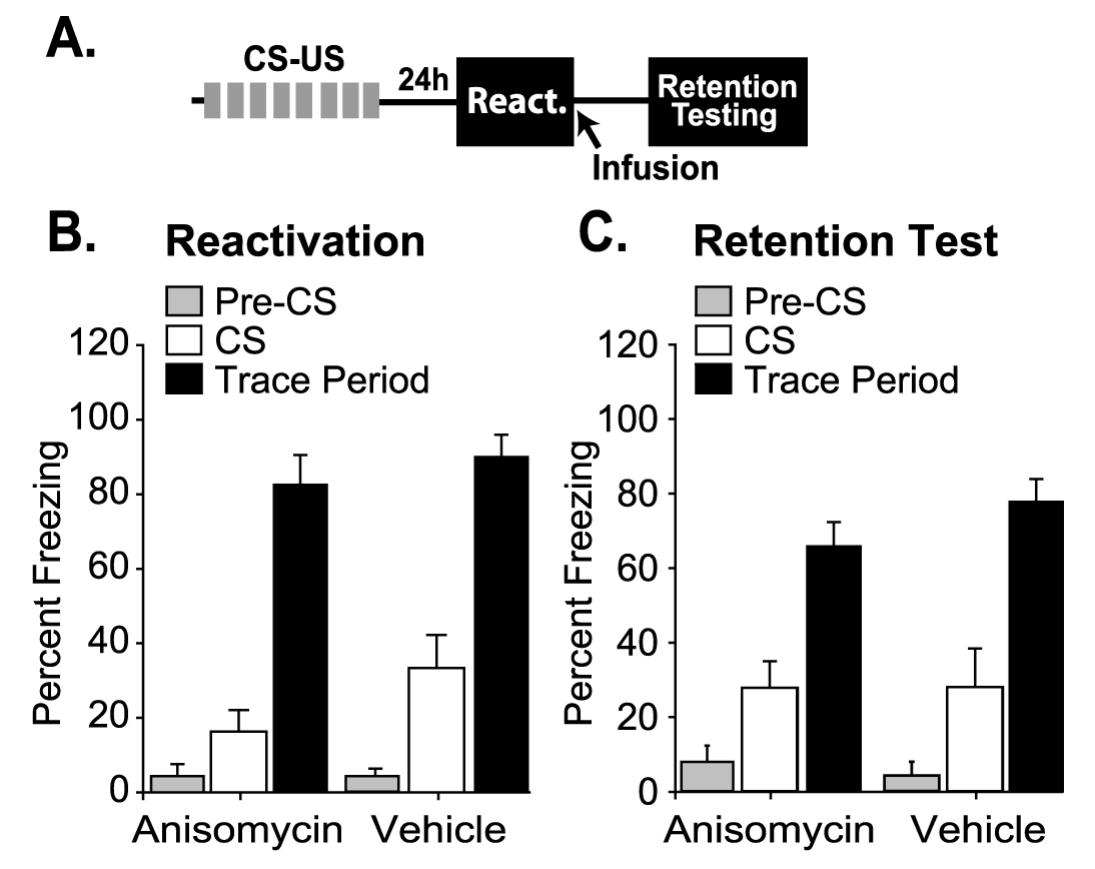
**Inhibition of prefrontal protein synthesis following recall of a one-day trace fear conditioning paradigm does not disrupt memory**. **A**, Scheme illustrating the chronology of training (CS-US), reactivation 24 hours later, intra-mPFC infusion, and retention testing in a novel context two days following the infusions. **B**, Graph of the mean percent freezing ± standard error of the mean (SEM) before CS presentation, during CS presentation, and during the trace period of the reactivation trial. Animals were infused with 2 ul of either anisomycin or vehicle directly into the mPFC, immediately following the reactivation trial. **C**, Graph of the mean ± SEM percent freezing before CS presentation, to the CS, and during the trace period of the retention testing trials for intra-mPFC anisomycin- and vehicle-infused groups, expressed as an average of four trials.

Animals were given a one-day training session consisting of eight CS-US paired trials (Fig. 1A). A 24-hour time point for reactivation has been previously used to show hippocampal reconsolidation for contextual, and trace fear conditioning [18,19]. Therefore, the animals were presented with one CS-alone reactivation trial within a novel context, 24 hours following training (Fig. 1A). Immediately following the memory reactivation trial, animals received bilateral intra-mPFC infusions of either 250 μg of anisomycin, or an equal volume (2 μl) of vehicle. The amount of anisomycin used in this study has previously been shown to inhibit >90% of protein synthesis, and is sufficient to impair consolidation [20,21] Additionally, infusion of this dose into the hippocampus was sufficient to observe reconsolidation of contextual [19], and trace fear conditioning [18].

During the reactivation trial, the animals demonstrated freezing behavior at a level comparable to the level acquired during training. Minimal freezing was observed in the novel context prior to CS presentation (pre-CS) and during CS presentation. A significant increase in freezing behavior during the trace period (Fig. 1B). Immediately following the reactivation trial, animals were divided into two comparable groups based on freezing behavior during the trace period of the reactivation trial, and one group was bilaterally infused with anisomycin (250 ug/2 ul per mPFC), while the other received vehicle (2 ul per mPFC) (trace period freezing: anisomycin n = 10: *react *= 82.5 ± 8.0 %; vehicle n = 9: *react *= 90.0 ± 6.0 %; n.s.). Retention was tested two days following reactivation by exposing the animals to four CS-alone trials and measuring trace period freezing. The two groups both remembered that the termination of the CS predicts the subsequent footshock as demonstrated by an increase in fear during the trace period as compared to that seen in either the pre-CS or the CS periods. However, the freezing percentage recorded for the anisomycin-infused animals during these time periods did not differ from that observed in the saline-treated controls (trace period freezing: anisomycin n = 10: *retention *= 65.8 ± 13.0 %; vehicle n = 9: *retention *= 77.8 ± 11.3 %; n.s.) (Fig. 1B–C), indicating no disruption in memory due to the protein synthesis inhibition. Contextual fear, assessed by placing the animal back into the original training chamber, also did not differ between the two groups (data not shown).

### Inhibition of prefrontal protein synthesis following recall of a two-day trace conditioning paradigm does not disrupt memory

**Figure 2:**
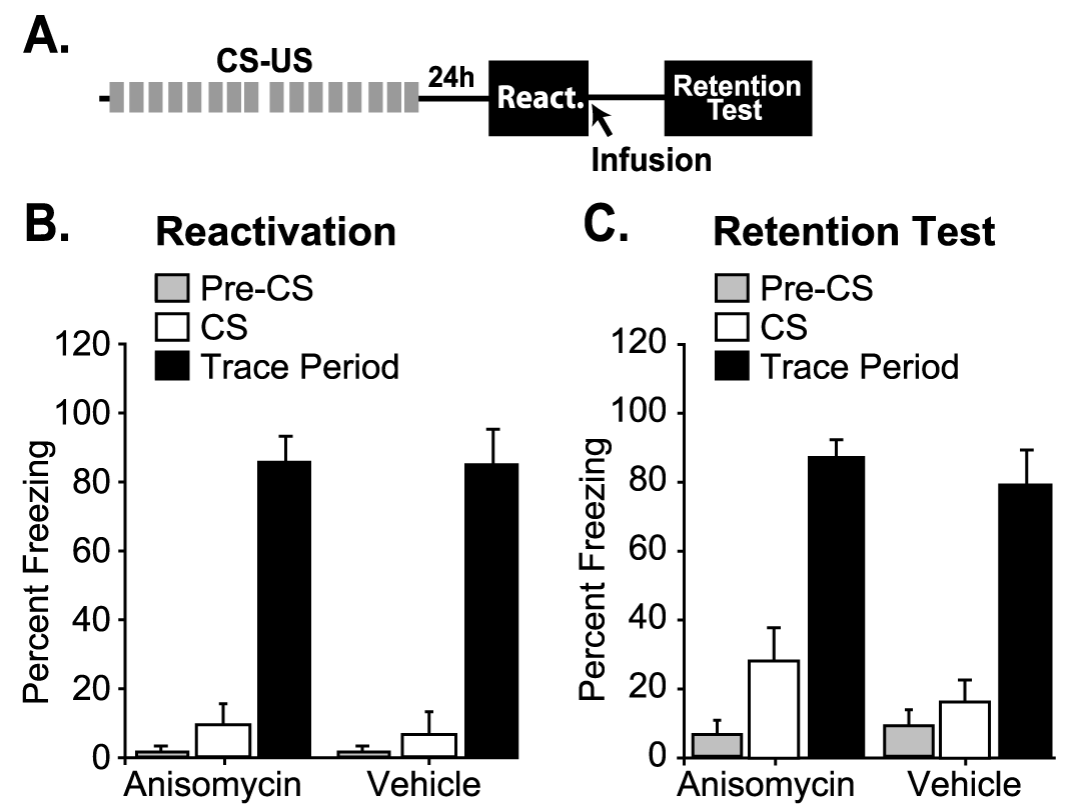
**Inhibition of prefrontal protein synthesis following recall of a two-day trace conditioning paradigm does not disrupt memory**. **A**, Scheme illustrating two training sessions (eight CS-US pairings on two consecutive days), reactivation 24 hrs following the second day of training, intra-mPFC infusion, and retention testing in a novel context. **B**, Summary data of the percent freezing during the pre-CS, CS and trace periods of the reactivation trial for intra-mPFC anisomycin- and vehicle-infused groups. **C**, Percent freezing displayed during the retention testing trials (pre-CS period, to the CS, and during the trace periods) for intra-mPFC anisomycin- and vehicle-infused groups. Data is expressed as the mean of four trials ± SEM.

We have previously shown that two consecutive days of trace fear training results in a memory which is susceptible to reconsolidation within the hippocampus [18]. To test if this protocol influences the susceptibility of the mPFC to protein synthesis inhibition following reactivation of trace fear memory, animals were given two eight-trial training sessions over two consecutive days. Twenty-four hours following the second training session, animals were given one CS-alone reactivation trial in a novel context, divided into two comparable groups, and bilaterally infused with either anisomycin (250 ug/2 ul per mPFC) or vehicle (2 ul per mPFC) (Fig. 2A–B). Retention was tested two days following reactivation by exposing the animals to four CS-alone trials and measuring trace period freezing. As observed in the one-day training paradigm, but in contrast to that previously observed in the hippocampus, the groups did not differ from each other in freezing during the trace period, with both groups displaying memory comparable to that during the reactivation trial (trace period freezing: anisomycin: *retention *= 87.14 + 5.22, vehicle: *retention *= 79.17 + 10.20, n.s.) (Fig. 2C). No difference in percent freezing between the groups was detected in either pre-CS or CS periods (Fig 2C), nor in context-specific fear (data not shown).

### Inhibition of prefrontal protein synthesis following remote recall of a two-day trace fear conditioning paradigm does not disrupt memory

**Figure 3:**
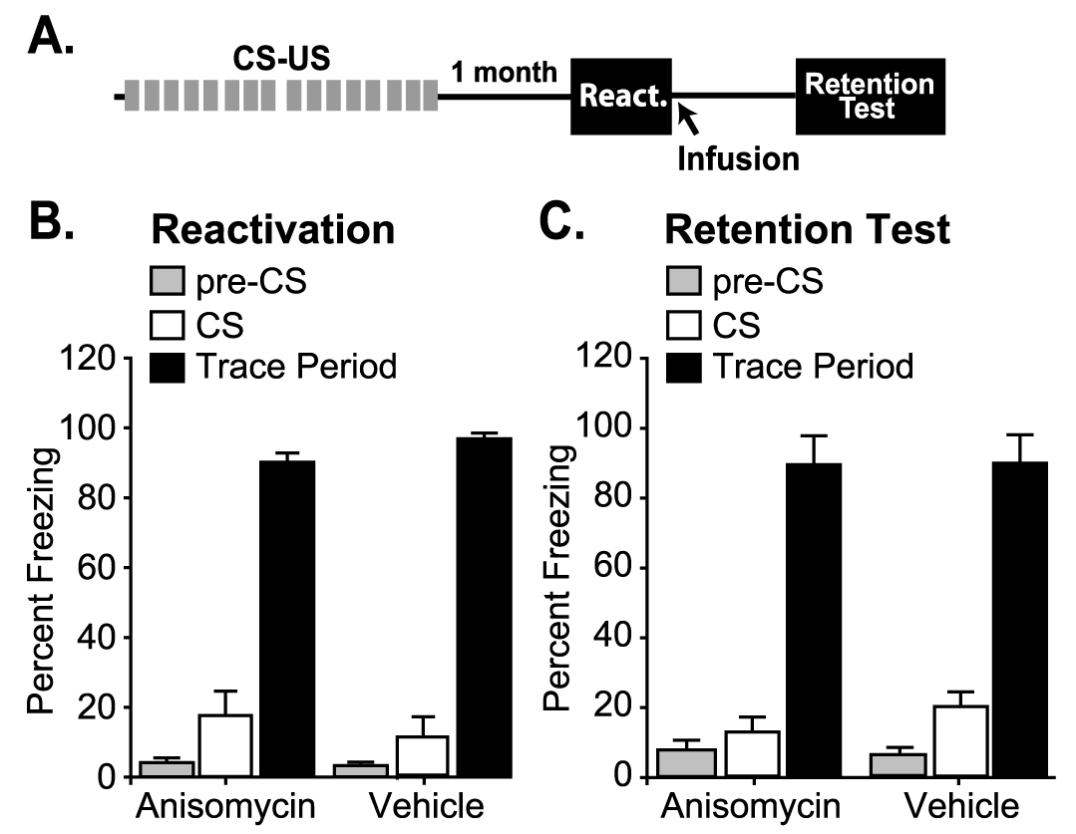
**Inhibition of prefrontal protein synthesis following remote recall of a two-day trace fear conditioning paradigm does not disrupt memory**. **A**, Scheme illustrating the chronology of training sessions on two consecutive days (eight CS-US per day), reactivation at 30 days following training, intra-mPFC infusion, and retention testing in a novel context (two days following the infusions). **B**, Graph of the mean ± SEM percent freezing prior to CS presentation, during the CS, and during the trace period of the reactivation trial for intra-mPFC anisomycin- and vehicle-infused groups. **C**, Graph of the mean ± SEM percent freezing during the pre-CS, CS and trace periods for the retention testing trials, expressed as an average of four trials.

Models of hippocampus-dependent memory consolidation posit that memory is initially dependent on the hippocampus, and remote memories are supported by neocortical regions (such as the mPFC) independently of the hippocampus [22-24]. The time scale of the switch from hippocampal to neocortical dependency is thought to be several weeks to a month in rodents [25-27] Although it has been recently demonstrated that memory storage within the mPFC occurs as a direct result of trace fear conditioning [16], it is still possible that reactivation of this memory at a time point beyond hippocampal dependency may be required to observe reconsolidation in this brain region. To test whether reactivation of remote memories induces protein synthesis dependent reconsolidation, animals were given eight CS-US pairings, on two consecutive days (Fig. 3A). Thirty days following training, memory was reactivated by exposing the animals to one CS-alone reactivation trial in a novel context (Fig. 3A). Immediately following the reactivation trial, animals were divided into two comparable groups (anisomycin: *react *= 90.0 ± 2.7%; vehicle: *react=*96.7 ± 1.7%, n.s.) (Fig. 3B), then bilaterally infused into the mPFC with either anisomycin or vehicle. Retention was tested two days following reactivation (32 days after training) by exposing the animals to four CS-alone trials and measuring trace period freezing. There was no difference between the groups during the retention trial in either the pre-CS, CS, or trace periods (trace period freezing: anisomycin: *retention *= 74.4 + 6.9%; vehicle: *retention *= 74.7 + 6.8%, n.s.) (Fig. 3C). No difference between the two groups in contextual fear was observed (data not shown).

**Figure 4:**
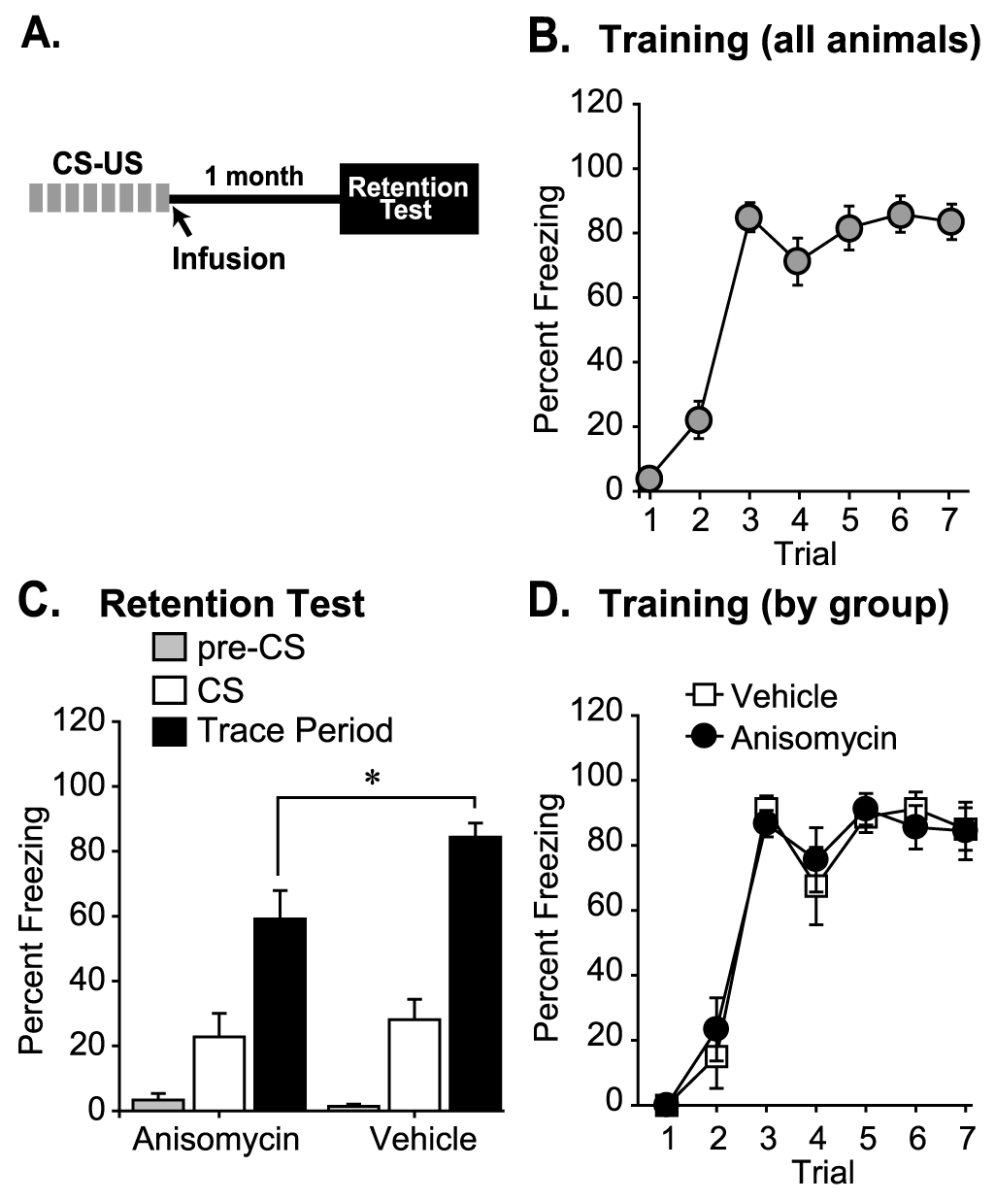
**Inhibition of prefrontal protein synthesis following trace conditioning impairs consolidation**. **A**, Scheme illustrating the training, intra-mPFC infusion, and retention testing in a novel context 30 days following the training. **B**, Summary data showing the learning curves for animals during trace fear conditioning. Data is presented as the mean ± SEM for percent freezing during the trace period. **C**, Percent freezing during the pre-CS, CS and trace periods of the reactivation trial for intra-mPFC anisomycin- and vehicle-infused groups. **D**, Summary data showing the learning curves of the vehicle- and anisomycin-infused animals during the trace conditioning paradigm. Data is presented as the mean ± SEM for percent freezing during the trace period. Data is presented as the mean ± SEM. *, P < 0.05.

Although the dose of anisomycin used in the present study has been employed by several laboratories, including ours, to evaluate reconsolidation, the absence of a reconsolidation effect in the mPFC may have resulted from insufficient protein synthesis inhibition. As consolidation has been repeatedly demonstrated to be dependent on *de novo *protein synthesis, the influence of anisomycin on mPFC memory storage was examined. Animals were trained in trace fear conditioning (Fig 4A). Figure 4B shows that animals acquired the conditioned repsonse as in indicated by increased freezing during the trace period. Immediately after completion of training, rats were randomly divided into two groups and infused with either anisomycin (at a dose of 160 μg per side), or an equal volume of vehicle directly into the mPFC. Thirty days following the training and infusion, retention of remote trace fear memory was tested by examining percent freezing in response to four cue presentations. Figure 4C shows that both groups displayed similar levels of freezing to the novel context (pre-CS) and to the CS. However, a significant reduction in freezing during the trace period was observed in the anisomycin-infused group by comparison to the vehicle-infused controls [*vehicle *(n = 8): 87.86 + 2.91; *anisomycin *(n = 10): 63.00 + 8.75; p < 0.05], (Fig 4C). This difference in trace fear could have arisen from a fortuitous sorting of the animals prior to anisomycin or vehicle infusion. To address this possibilty, we re-examined the acquisition curves of the conditioned animals separated into their respective treatment groups. Figure 4D shows that there was no observable difference in the learning curves between the two groups. Taken together, these results demonstrate that anisomycin infusion into the mPFC impairs memory consolidation for trace conditioning, but that these memories do not appear to undergo reconsolidation.

**Figure 5:**
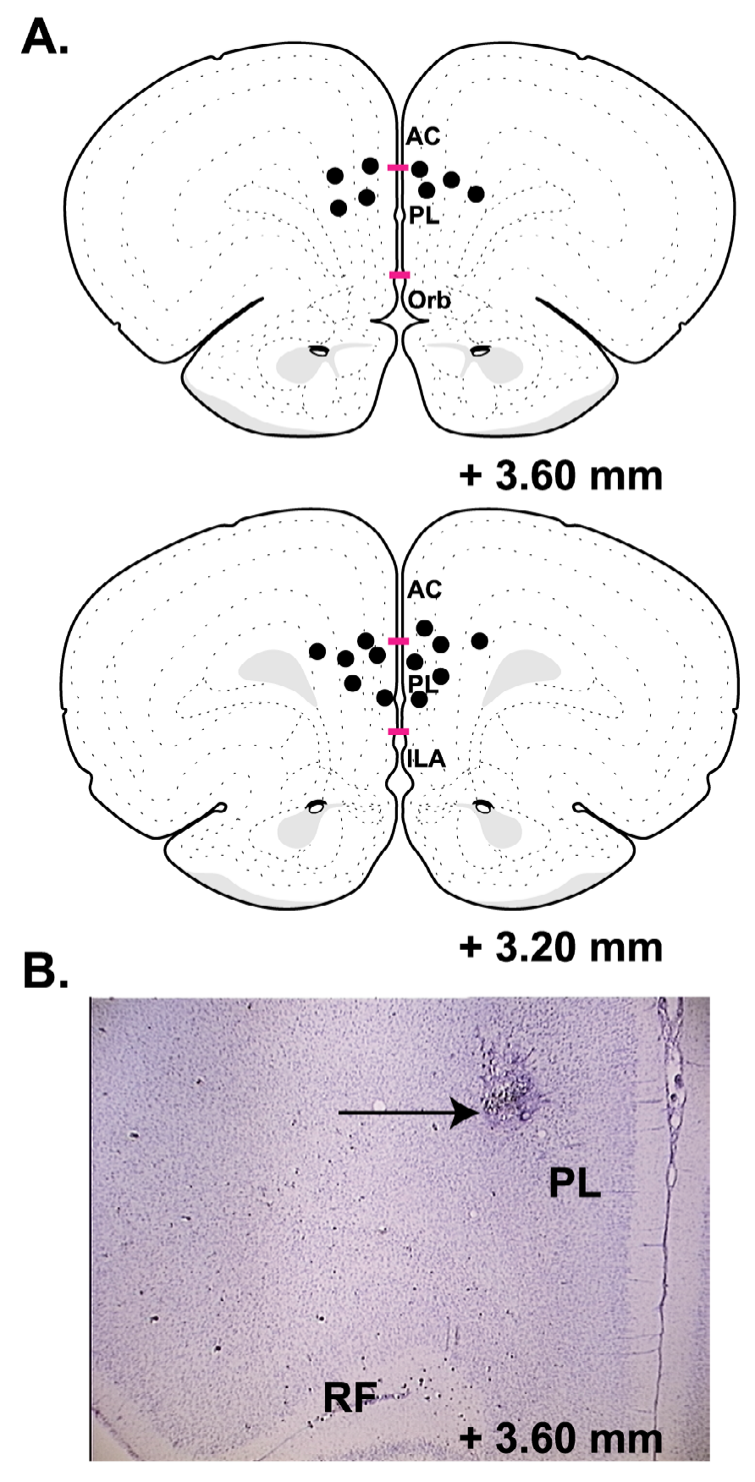
**Sites of intra-mPFC anisomycin infusions**. **A**, Depiction of the mPFC on atlas plates at two different rostral-caudal planes modified from Swanson (2004). Non-redundant sites of infusion termini (marked by black circles) in the mPFC from a representative sample of animals. The horizontal bars mark the borders of the prelimbic (PL) cortex. None of the infusion tracks terminated in the infralimbic (IL) cortex. **B**, A representative photomicrograph of a coronal section stained with cresyl violet indicating the terminus of an infusion needle (shown by arrow). AC, anterior cingulate; ILA, infralimbic cortex; PL, prelimbic cortex; RF, rhinal fissure; Orb, medial orbital cortex.

Following completion of behavioral studies, representative animals were examined for cannulae placement. The infusion track termini were located in the PL cortex of the mPFC in the animals examined (Fig. 5A–B). All of the infusion sites were located within 0.35 mm (along the raustro-caudal axis) of the target coordinates.

## Discussion

Reconsolidation refers to the experimental observations that following reactivation, a memory trace may once again become susceptible to protein synthesis inhibition [3,9,19] Although this is similar to the protein synthesis dependency of initial stabilization or consolidation of memory, one important distinction is that not every brain region involved in consolidation is involved in reconsolidation [9-11] In trace fear conditioning, memory for the trace relationship is stored in both the hippocampus and mPFC [16,28] We have recently demonstrated that inhibition of hippocampal protein synthesis following reactivation of trace fear memory results in reconsolidation [18]. In contrast, the present study demonstrates that memory for trace fear conditioning does not undergo protein synthesis-dependent reconsolidation in the mPFC, regardless of the intensity of the training, and regardless of whether the memory is recent or remote.

As we are reporting that we find no evidence of reconsolidation in the mPFC, we have to consider the possibility that this negative finding is due to an insufficient dose of the protein synthesis inhibitor. Effects in reconsolidation studies have previously been observed in structure-targeted infusions of as little as 62.5 μg of anisomycin [3]. In the present study, we infused four times this amount (250 μg), a dose that has previously been shown to result in reconsolidation when infused into the hippocampus immediately following reactivation [18,19]. Furthermore, a dose of 160 μg anisomycin, two-thirds that used in the reconsolidation experiments, was sufficient to impair long-term consolidation within the mPFC. It is therefore unlikely that insufficient protein synthesis inhibition underlies the absence of a reconsolidation effect. Several studies have suggested that specific reactivation conditions may be required to induce reconsolidation in some situations [29,30] For example, in an object recognition task, reactivation of memory in the original context is required in order to observe *zif268 *dependent reconsolidation [30]. Although the present study did not identify the specific conditions, if any, required for reconsolidation in the mPFC, it demonstrated that the paradigm that induces this phenomenon in the hippocampus is not capable of causing reconsolidation in the mPFC.

At present, it is still debated whether reconsolidation is analogous to a recapitulation of initial consolidation, if it reveals a late component of the initial consolidation process, or if it is related to memory retrieval (for review see [31])[5,6]. Recently, it has been shown that impaired memory as a result of inhibition of hippocampal protein synthesis following memory recall can be rescued by a reminder).)[32]. This, along with studies demonstrating that memory impairments as a result of hippocampal reconsolidation reverse over time [4,33]., suggests that the memory deficits may result from a dysfunction in memory retrieval. The mPFC has several characteristics which may explain the lack of reconsolidation in this structure, regardless of whether reconsolidation reflects memory storage or retrieval deficits. First, if we assume that reconsolidation is reflective of a memory storage deficit, the mPFC may serve a different function in memory storage than the hippocampus. Most models of long-term memory consolidation for hippocampus-dependent memories, such as trace fear conditioning, posit that the hippocampus plays a temporary role, and that remote, long-lasting memories are dependent on neocortical areas such as the mPFC. Although long-term plasticity is observed in both the mPFC and the hippocampus as a direct result of training, this does not imply that the memories stored in these structures are of equivalent strength. It is possible that plasticity within the mPFC is more stable, and not susceptible to reconsolidation, allowing for persistence of memory over months to years. Second, if reconsolidation reflects a retrieval deficit, the absence of reconsolidation in the mPFC may reflect the difference between the roles played by the hippocampus and the mPFC in this process. One possibility is that information storage within the hippocampus may be involved in the reactivation of various components of information stored in other structures and memory reactivation results in protein synthesis-dependent modifications within the hippocampus that are necessary for subsequent memory retrieval of updated memories [5]. In contrast, in the trace fear conditioning paradigm, the mPFC may be involved in storing some aspect of the information that is retrieved and, therefore, may not undergo reconsolidation. Finally, we would like to point out that despite the fact that anisomycin has been the most widely used protein synthesis inhibitor, both in consolidation and reconsolidation experiments (for review see [2])[34], this inhibitor has a number of non-specific effects, including apoptosis [35,36]. At present, it is not known if these non-specific influences may have contributed to the observation of reconsolidation in the hippocampus, and its lack in the mPFC. As more insight is gained into the processes underlying the observations of reconsolidation, it should become possible to determine the differences between the hippocampus and the mPFC in respect to their role in reconsolidation.

## Conclusion

The present study demonstrates three key findings: 1) trace fear memory does not undergo protein synthesis dependent reconsolidation in the PFC, regardless of the intensity of the training, and 2) regardless of whether the memory is recent or remote, and 3) intra-mPFC inhibition of protein synthesis immediately following training impaired remote (30 days) memory.

## Methods

### Subjects

Male Sprague Dawley rats (Charles River Laboratory, Wilmington, MA) weighing 250 to 300 gm were pair-housed under temperature-controlled conditions with a 12 hr light/dark cycle. All rats were given *ad libitum *access to water and food. Protocols regarding the training and surgery of the animal subjects were approved by the Institutional Animal Welfare Committee and were in compliance with NIH's *Guide for Care and Use of Laboratory Animals*.

### Surgery

Animals were anesthetized under 5% isoflurane with a 2:1 N_2_O/O_2 _mixture and then maintained under 2.5% isoflurane with a 2:1 N_2_O/O_2 _mixture via a face mask. Twenty-two gauge stainless-steel guide cannulae were implanted into the mPFC (bregma + 3.2 mm, lateral ± 0.75 mm, and depth -2.5 mm) using a stereotaxic device [37]. Animals were given a ten-day rest period following surgery before behavioral testing. For the 30 day reconsolidation study, animals were trained in the trace fear conditioning task first, and the guide cannulae were implanted ten days prior to the reactivation/reconsolidation testing. This was done to prevent excessive scar tissue formation, and drift of the guide cannulae, which may occur over a one month period. Infusion cannulae that extended 1.5 mm past the end of the guide cannulae were used for drug infusions. After the completion of all behavioral experiments, representative animals were killed and the brains post-fixed in a 4% paraformaldehyde solution for histological analysis of cannulae location.

### Pharmacological infusions

Anisomycin (Sigma, St. Louis, MO) was dissolved in 1 N hydrochloric acid, neutralized with NaOH, and then diluted in artificial cerebrospinal fluid (ACSF), pH 7.4. Anisomycin was bilaterally infused into the mPFC (250 μg/2 μl per side for reconsolidation; 160 μg/side for consolidation), at a rate of 0.25 μl per minute. The dose of 250 μg/2 μl has previously been shown to be effective in blocking reconsolidation [18,19]. Anisomycin was infused immediately following a single CS-alone exposure in a novel context (reactivation trial). Vehicle control infusions consisted of the same volume ACSF with the same pH as the anisomycin solution. Following the infusion, the needle was left in place for two minutes to allow for diffusion of the drug. All infusions were performed using a motorized infusion pump (Stoelting, Wood Dale, IL).

### Trace fear conditioning/recall

All behavioral tests were performed by an investigator who was blind to the treatment groups. Animals were placed in the training context (*Habitest Unit*, Coulbourn Instruments, Allentown, PA) and given a 120 second habituation period. Conditioning trials began with a 10 second tone (CS) followed by a 20 second trace period, after which the animal received a 0.8 mA foot-shock (US) for 0.7 seconds. Each CS-US paired training trial was separated by a pseudorandom inter-trial interval (ITI) that varied between 1–4 minutes. A pseudorandom ITI was used so that amount of time between foot-shocks could not be used as a cue for the US. For reactivation of memory, animals were placed in a novel context, given a 120 second habituation period followed by one presentation of the CS without the presentation of the US (CS-alone reactivation trial). Immediately following the reactivation trial, animals were divided into two comparable groups based on freezing behavior during the trace period of the reactivation trial. One group was bilaterally infused with anisomycin and the other with vehicle. For retention testing, each animal was placed in a novel context and given a 120 second habituation period. In the absence of foot-shock, four presentations of the CS were given separated by a varied ITI period. During the retention and reactivation trials, freezing behavior (defined as the absence of all movement excluding movement caused by respiration) was measured during the CS, trace, and ITI periods. Freezing behavior was recorded every two seconds during scoring periods. Freezing behavior during the four CS presentations was averaged for each animal. Following trace CS-US retention testing; contextual retention was measured by placing the animals back into the original training context for a 90 sec period during which freezing was scored, without exposure to the CS or US.

### Statistics

A two-way repeated measures *ANOVA *was used to compare the percentage of trace period freezing during retention testing between the anisomycin- and vehicle-infused groups as well as to compare trace period freezing between reactivation and retention trials, within and between the groups. An unpaired *t-test *was used to compare CS as well as contextual freezing between the anisomycin- and vehicle-infused groups. A *p *value of ≤ 0.05 was used as the criterion for statistical significance. Raw data were used for all statistical analyses.

## Abbreviations

CS, conditioned stimulus; CTA, conditioned taste aversion; iti, intertrial interval; mPFC, medial prefrontal cortex; PL/IL, prelimbic/infralimbic; US, unconditioned stimulus.

## Authors' contributions

SB participated in the design of the study, carried out behavioural experiments, histological analysis, statistical analysis, and drafted the manuscript. JR participated in the design of the study, carried out behavioural experiments and statistical analysis. PD participated in the design of the study, coordinated the study, and revisions of the manuscript.
